# Good Gig, Bad Gig: Autonomy and Algorithmic Control in the Global Gig Economy

**DOI:** 10.1177/0950017018785616

**Published:** 2018-08-08

**Authors:** Alex J Wood, Mark Graham, Vili Lehdonvirta, Isis Hjorth

**Affiliations:** University of Oxford, UK; University of Oxford, UK; University of Oxford, UK; University of Oxford, UK

**Keywords:** Flexibility, gig economy, job quality, labour process, platform economy, workplace control

## Abstract

This article evaluates the job quality of work in the remote gig economy. Such work consists of the remote provision of a wide variety of digital services mediated by online labour platforms. Focusing on workers in Southeast Asia and Sub-Saharan Africa, the article draws on semi-structured interviews in six countries (*N* = 107) and a cross-regional survey (*N* = 679) to detail the manner in which remote gig work is shaped by platform-based algorithmic control. Despite varying country contexts and types of work, we show that algorithmic control is central to the operation of online labour platforms. Algorithmic management techniques tend to offer workers high levels of flexibility, autonomy, task variety and complexity. However, these mechanisms of control can also result in low pay, social isolation, working unsocial and irregular hours, overwork, sleep deprivation and exhaustion.

## Introduction

The ‘gig economy’ has emerged as a key theme in a recent independent review of modern employment practices (Taylor et al., 2017). The review was commissioned by the UK government in response to the perceived growth of precarious work. The review defined the gig economy as ‘people using apps [also commonly known as platforms] to sell their labour’ (Taylor et al., 2017: 23). In Britain, Lepanjuuri et al. (2018) find that 4.4% of adults have worked in the gig economy in the last year and suggest that 2.4% of adults do so at least monthly. The gig economy consists both of work that is transacted via platforms but delivered locally and thus requires the worker to be physically present, and work that is transacted and delivered remotely via platforms (Huws et al., 2016). Local gig work includes food delivery, couriering, transport and manual labour. Remote gig work by contrast consists of the remote provision of a wide variety of digital services, ranging from data entry to software programming (see Table 1), via platforms such as Amazon Mechanical Turk (MTurk), Fiverr, Freelancer.com and Upwork. Though locally delivered gig work has received much attention, surveys suggest that the remote type makes up a similar proportion of the UK gig economy (Chartered Institute of Personnel Development (CIPD), 2017; Huws et al., 2016; Lepanjuuri et al., 2018). Globally, 70 million workers are estimated to have registered with online labour platforms that facilitate remote forms of gig work (Heeks, 2017). An index measuring the utilisation of online labour platforms suggests that their use is growing at an annual rate of 26% (Kässi and Lehdonvirta, 2016).

**Table 1.** Online gig work (reproduced from Kässi and Lehdonvirta, 2016).

Occupation class	Examples of projects
Professional services	Accounting
	Consulting
	Financial planning
	Human resources
	Legal services
	Project management
Clerical and data entry	Customer service
	Data entry
	Tech support
	Transcription
	Virtual assistant
	Web research
Creative and multimedia	Animation
	Architecture
	Audio
	Logo design
	Photography
	Presentations
	Video acting
	Video production
Sales and marketing support	Ad posting
	Lead generation
	Search engine optimization
	Telemarketing
Software development and technology	Data science
	Game development
	Mobile development
	QA and testing
	Server maintenance
	Software development
	Web development
	Web scraping
Writing and translation	Academic writing
	Article writing
	Copywriting
	Creative writing
	Technical writing
	Translation

Although the absolute number of workers in the gig economy remains relatively small, there is concern among the public and policy makers in high-income countries regarding its implications for the future of work. For instance, it has been argued to fragment work, increase casualisation and undermine the standard employment relationship (De Stefano, 2016). Among policy makers in lower- and middle-income countries and institutions concerned with fostering economic development, the growth of gig work has generally been received more positively. A range of initiatives have been created with the aim of capitalising on the opportunities presented by online outsourcing and remote gig work in order to bring millions of jobs to potential workers in lower-income countries (The Rockefeller Foundation, 2013; United Nations Development Programme (UNDP), 2016; World Bank, 2016). However, research on the quality of remote gig work remains limited, both in the number of studies undertaken and breadth of platforms and countries investigated (D’Cruz and Noronha, 2016).

In the context of limited scholarship on the topic and the growth of the gig economy, this article makes two important contributions. First, it identifies commonalities in the ‘job quality’ of remote gig work across various national contexts and types of work. Second, it provides a theoretically informed explanation for these outcomes. The article uses 107 semi-structured interviews and a survey of 679 Southeast Asian and Sub-Saharan African workers to identify common themes in the experiences of online gig workers. This provides us with data from workers undertaking a range of tasks within differing national contexts. The existence of commonalities in job quality despite the heterogeneity of our sample is surprising, but can be explained by technology and power producing regularities in worker–platform, worker–client and worker–worker relations. Our analysis highlights the importance of algorithmic control systems, labour oversupply and high levels of competition between workers for job quality outcomes.

## The growth of market-mediated, open employment relations

Kalleberg (2011) highlights the growth of ‘market-mediated, open employment relationships’ in the United States.^1^ This form of employment relationship entails the replacement of administrative rules with market mechanisms for determining job outcomes. It also entails the shifting of economic risks and responsibility for skill development on to workers, as firms are no longer willing to provide security and training for their workforces. Market-mediated, open employment relationships are ‘based on free market forces and competition and are associated with relatively weak labour market institutions, standards and regulations’. They can be contrasted with closed employment relations based on strong institutional protections ‘derived from unions or firm internal labour markets’ (Kalleberg, 2011: 83). In high-income countries, the growth of the gig economy can be situated within this context and has thus been seen as a threat to the closed employment relations which dominated the post-war period; being equated with the fragmentation, casualisation and precarisation of work in high-income countries (De Stefano, 2016; Standing, 2016).

Webster et al. (2008) document how closed employment relations also grew in low- and middle-income countries during the post-war period. Forms of state corporatism developed as the labour movements which often accompanied national liberation struggles were integrated by new post-colonial states. Workers in the formal economy were granted certain legal rights and guarantees over labour conditions, even as the majority of workers were excluded from this institutionalisation due to their location in a parallel informal economy. Early in the 1990s, state corporatism started to come under pressure, usually as a result of International Monetary Fund structural adjustment programmes aimed at achieving ‘labour market flexibility’ (Webster et al., 2008: 54). The resulting expansion of market-mediated, open employment relationships has been received less negatively in the low- and middle-income countries, perhaps because the majority of workers never had a chance to experience or benefit from closed employment relations in the first place.

The growth of online labour platforms in low- and middle-income countries has been seen as enabling a new wave of online outsourcing entailing employment growth and poverty reduction (The Rockefeller Foundation, 2013; UNDP, 2016; World Bank, 2016). Yet little is known about the job quality of the new opportunities being created.

## Job quality and remote gig work

The concept of job quality was originally developed in high-income countries and its application to lower- to middle-income countries has been limited, due to both paucity of data and the failure of the International Labour Organisation’s (ILO) Decent Work programme (Burchell et al., 2014). However, there are some instances of studies investigating job quality in low- and middle-income countries, and the core dimensions are considered to be transferable (Burchell et al., 2016; Monteith and Giesbert, 2017). As Rubery and Grimshaw (2001: 167) point out, job quality is not only a concern for ‘highly-skilled jobs or rich countries. It also relates to jobs at the low-skill end of the spectrum in every society.’ Monteith and Giesbert (2017) use focus groups to investigate perceptions of job quality among informal sector workers in Uganda, Burkina Faso and Sri Lanka. They find that these workers value a number of the same features which have been considered important for job quality in high-income countries, such as income, health, autonomy, control over work activities and hours, and social contact.

One of the few detailed empirical investigations of remote gig work is Berg’s (2016) survey of 1510 workers on MTurk and CrowdFlower. Berg (2016) finds low pay to be a problem, and that major contributors to low pay are the high ratio of unpaid work to paid labour and a lack of available work. Workers without alternative employment (around 40% of Berg’s sample) lacked employment-linked social security (Berg, 2016). However, Berg’s findings also highlight some positives. Workers controlled their place of work, which enabled the overcoming of barriers to labour market participation. Although these findings are important, their ability to illuminate the job quality of remote gig work more generally is limited. Both MTurk and CloudFlower are ‘microwork’ platforms, which by definition provide the most fragmented, deskilled and commodified work (Bergvall-Kåreborn and Howcroft, 2014; Lehdonvirta, 2016). MTurk’s workforce is also geographically concentrated; recent surveys suggest that as much as 80% of the workers are located in the United States and 16% in India (Hitlin, 2016). MTurk and Crowdflower are thus in many ways not representative of wider remote gig work.

The world’s largest platform for remote gig workers, Upwork, hosts both microwork and more skilled job tasks. This platform was the focus of a study by D’Cruz and Noronha (2016), who demonstrated that workers on the platform benefit from being able to control their place of work. The authors also identify some negatives, including high levels of uncertainty – though this was argued to result from part-time workers ‘having restricted their efforts to enhance their positive experiences’ (D’Cruz and Noronha, 2016: 51). Like their MTurk and CrowdFlower counterparts, D’Cruz and Noronha (2016) find that Upwork workers undertake substantial unpaid work in order to get paid work, and this invariably meant working at night. The competitive work organisation, whereby all workers can view each other’s bids, was also found to be a source of downward pressure on pay rates. However, these findings are based upon telephone interviews with just 24 Indian workers. Existing research on the quality of online gig work is clearly limited, in terms of both the number of studies undertaken and the breadth of platforms and countries investigated. The initial aim of this article is to rectify these limitations by elucidating whether commonalities in job quality can be identified across two large remote gig work platforms and across six underexplored national contexts.

### Explaining job quality

A further issue with the extant remote gig economy literature is that it makes little attempt to explain job quality outcomes. Rubery and Grimshaw (2001: 165) argue that an important determinate of job quality is the ways in which information-communication technologies (ICTs) affect organisational forms, and that this will be critically shaped by the balance of bargaining power between employer and employee. Likewise, Mullan and Wajcman (2017) argue that the relationship between work-related time pressure and digital technology is mediated by work arrangements. Additionally, Silver (2003) demonstrates that once adopted particular technologies impact on worker power in specific and often unintended ways. Worker power refers to ‘the ability of employees, collectively or individually, to obtain an advantaged position in the stratification system’ (Kalleberg, 2011: 31). Kalleberg (2011: 83) argues that in market-mediated employment relations, ‘the centrality of market mechanisms makes individuals’ resources […] more vital [than collective] sources of power’. Therefore, it is expected that ‘marketplace bargaining power’ – labour demand, skills and alternative non-wage sources of income (Silver, 2003; Wright, 2000) – will be key in determining job quality for remote gig workers.

**Figure 1.**
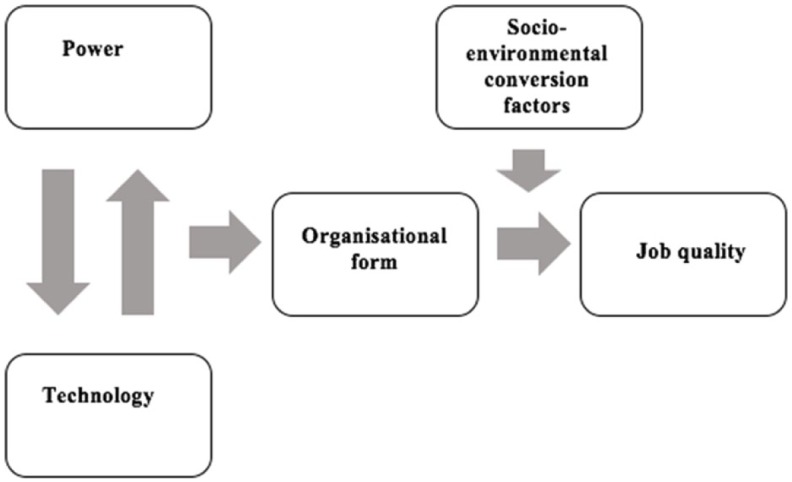
A model of job quality determinants.

It is important to recognise that workers do not only exert power relative to employers, but also relative to other workers (Kalleberg, 2011). Workers with differing levels of marketplace bargaining power are therefore expected to have divergent job quality experiences. Additionally, Monteith and Giesbert (2017) argue that the ability of work to provide particular capabilities and benefit well-being is dependent on what they term social and environmental conversion factors. Taking these conversion factors into account is held to be of particular benefit to job quality studies in informal, developing country contexts. In particular, Monteith and Giesbert (2017) highlight the mediating effects of health and educational systems, gender norms and relations. These insights are combined in Figure 1.

### Digital control

Concerning the impact of technology on organisational forms, many sociological accounts of ICTs, in both high-income and lower-income countries, emphasise the possibilities they entail for high levels of monitoring and measurement of work (Taylor and Bain, 2005; Woodcock, 2017). This is argued to represent ‘significant developments in the Taylorisation of white-collar work’ (Bain and Taylor, 2000: 9). This Taylorist informational control and discipline involves subjecting work tasks to detailed digital measurements and statistical analyses of individual worker performance. Underperforming workers face intense supervisory pressure and discipline on the basis of remote covert monitoring of their work. Such accounts equate the resultant organisational form of work to an ‘assembly line in the head’ (Bain and Taylor, 2000), or ‘electronic sweatshop’ (Fernie and Metcalf, 1998) in which high levels of technical and bureaucratic controls are combined (Callaghan and Thompson, 2001). In terms of job quality, this entails a lack of worker autonomy combined with high levels of work intensity.

Accounts of Taylorist informational control are predominately based on research in call centres. However, this research has informed more general theories regarding the adoption of technology being ‘effort biased’ (Rubery and Grimshaw, 2001). Empirical studies find that computerisation seemingly intensifies work by increasing monitoring, raising its pace, minimising the gaps between work tasks and extending work activity beyond the conventional workplace and working day (Felstead et al., 2016; Green, 2004). Longer working hours, higher work intensity and work–home spill-over have been found to be associated with remote working made possible by new digital technologies (Felstead and Henseke, 2017).

In the gig economy it has been argued that a particularly important form of digital control is the ‘algorithmic management’ entailed by platform-based rating and reputation systems. Algorithmic management is an extension of ‘customer management’ strategies, which entails positioning customers ‘as agents in the management circuit’, so that ‘customers, rather than managers, are […] the ones who must be pleased, whose orders must be followed, whose ideas, whims and desires appear to dictate how work is performed’ (Fuller and Smith, 1991: 11). Rosenblat and Stark (2016) demonstrate the effectiveness of Uber’s use of algorithmic management in structuring its workforce’s behaviour through ‘soft’ control.

The second aim of this article is therefore to identify the organisational forms (such as Taylorist and algorithmic controls) that could explain commonalities in the quality remote gig work across the platforms and countries that we study, and how these organisational forms are shaped by power and technology.

## Methods

Our analysis is primarily based on face-to-face semi-structured interviews with 107 workers. The worker informants consist of 36 workers in Southeast Asia (SEA, comprising 12 in the Philippines, five in Malaysia and 19 in Vietnam), and 71 in Sub-Saharan Africa (SSA, comprising 29 in Kenya, 23 in Nigeria and 19 in South Africa). The interviews were conducted during seven months of fieldwork in Southeast Asia and Sub-Saharan Africa (September 2014 to December 2015). In total 147 workers were interviewed, however, in the present analysis we exclude 40 workers who did not use either of the platforms discussed below. For an overview of the project, see Graham et al. (2017b). The sampling strategy was purposive, the main aims being to ensure that workers had been active on one of two leading platforms for at least six months, that a diversity of work types were covered and that there was a rough gender balance. Both platforms had millions of registered clients, offer a similarly broad range of freelance services and share comparable basic design features including a double auction structure whereby both workers and clients can suggest prices.

We recruited informants by posting hidden tasks on the online labour platforms. The task description provided information about the research project, questions relating to informed consent and clarified that participation was voluntary, and should not be seen as a job. To further make that point, it was emphasised that no feedback would be left as an outcome of the participation. Expenses for transport would be reimbursed, and participants would receive $6 as a token of gratitude. A relationship and trust were built with the informants before the interview so as to ensure that they genuinely perceived the interview situation as research participation rather than someone performing a paid task.

These qualitative data were coded, following Vaughan’s (1992) theory elaboration approach. Initial codes were informed by the extant job quality literature and new codes developed out of an iterative process. Nvivo enabled systematic theoretical coding to be undertaken and hundreds of initial codes to be generated. Focused coding was then employed to highlight the most common and revealing initial codes and to merge appropriate initial codes into new higher level codes, as suggested by Charmaz (2006).

We also present results from a non-probability survey of 679 workers in SEA and SSA. The survey results are used to demonstrate that themes developed from our interview informants’ experiences are also shared by many other remote gig workers in these countries. When survey results show significant differences between social groups or geographic areas, these are also presented.

For the purposes of survey research, the population of remote gig workers is an ambiguous concept, as people’s engagement with platforms varies from merely registering an account to earning a full-time income, and also changes over time. We decided to define the population as people in SEA and SSA who had completed work through one of the two leading platforms within the past two months and had in total worked in at least five projects or for five hours. We recruited the participants by posting the survey as a paid task worth $3. Probability sampling is not possible through an online labour platform (at least without the platform owner’s collaboration), because the probability of members seeing and responding to the survey task is unequal and unknown. Instead, we followed a purposive sampling strategy, where the platforms’ search features were used to identify a mix of potential participants across different countries, genders and skills. The potential participants were then personally invited to respond to the survey task. The result is similar to a stratified or quota sample, except that the subsample sizes were not predefined but depended on how easy or difficult it was to find members of each subpopulation on the platform. The result is likely to be more representative of the population than samples recruited simply by posting an open task because it mitigates self-selection biases from task type preferences and reservation wages. The response rates to the invitations were 30% and 7% on the two platforms respectively, which are low by conventional social survey standards, but an improvement on entirely self-selecting online surveys. We obtained a total of 853 responses, of which 174 were excluded due to not being located in SEA or SSA, not having completed the modules related to job quality or failing an attention check. This left us with a sample of 679 workers, of which 40% were female and 60% male and 51% were located in SEA and 49% in SSA.

## Findings and discussion

### Autonomy in the shadow of algorithmic management

An important theme that emerged from our interviews was the perceived autonomy and discretion that workers experienced. This finding contrasts with accounts which suggest ICTs lead to Taylorist organisational forms being applied to white collar work. In this case the possibility for direct observation and supervision of remote gig work was limited by the non-proximity of workers and clients. Our informants explained how their clients tended to be based in high-income countries while they themselves were based in SEA and SSA. This placed spatial and temporal constraints on clients’ ability to accurately monitor the labour process and issue instructions. As Mark (Kenya; writing, lead generation,^2^ internet advertisement tasks) summed up: ‘I don’t have someone supervising, telling you: “you have not done this, you have not done this,” yelling at you.’

Nevertheless, all economic exchanges require ‘systems of control’ (Granovetter, 2005; Wood, 2017) and as Rosenblat and Stark (2016: 3772) argue, ‘digital spaces facilitate and scaffold new systems of monitoring and opportunities for remote control over workers.’ Both platforms attempted to overcome barriers to labour process observation by providing non-proximate monitoring mechanisms. For instance, frequencies of workers’ keyboard presses and mouse movements were tracked and shots taken of their screens. However, these monitoring mechanisms were easily circumvented. Simon (Kenya; lead generation, internet advertisement, transcription) explained how he used timing to work around the screenshots: ‘It comes once every 10 minutes. Once it has shown up in a 10-minute block you leave… you have nine minutes to do everything that is totally non-related to work.’ The system could also be bypassed simply by setting up a second monitor: ‘Since I’m technical I connect my laptop to my TV… so I have two screens, I’m watching YouTube while I’m working on the platform… because the screenshot is only for the main [monitor]’ (Nicole, Philippines; virtual assistance, translation, writing). In fact, the monitoring was often welcomed by workers, because the platform guaranteed payments for monitored work, protecting workers from non-payment and wage theft (as also suggested by D’Cruz and Noronha, 2016).

A far more effective means of control was the ‘algorithmic management’ enabled by platform-based rating and reputation systems (Lee et al., 2015; Rosenblat and Stark, 2016). Workers were rated by their clients following the completion of tasks. Workers with the best scores and the most experience tended to receive more work due to clients’ preferences and the platforms’ algorithmic ranking of workers within search results. This form of control was very effective, as informants stressed the importance of maintaining a high average rating and good accuracy scores. Whereas Uber’s algorithmic management ‘deactivates’ (dismisses) workers with ratings deemed low (Rosenblat and Stark, 2016), online labour platforms, instead, use algorithms to filter work away from those with low ratings, thus making continuing on the platform a less viable means of making a living.

What is notable about this algorithmic system of control in terms of job quality, compared to Taylorist forms of informational control, is that control operated at the end of the labour process rather than during it. This afforded workers freedom to work however they wished as long as the end product was accurate or satisfactory to the client. In effect, this organisational form afforded significant autonomy and discretion. As Victor (Nigeria; web research, data entry, lead generation, transcription and translation) put it: ‘You have the freedom of choice. Who you want to work with, when you want to work, and how you want to work.’

Shapiro (2017) refers to the autonomy of local gig workers over things such as when to work and which orders to accept or reject as only constituting ‘autonomy over minute decisions’. Yet our findings suggest that for remote gig workers the level of discretion is far from minute. This is echoed in our survey, where 72% of respondents felt able to choose and change the order in which they undertook online tasks, and 74% were able to choose or change their methods of work. Customer management strategies often go hand-in-hand with worker empowerment (Fuller and Smith, 1991). The lack of standardisation of most remote gig work would make it difficult to institute Taylorist methods of control, and such methods would probably be self-defeating in extinguishing the discretion and spontaneity necessary for workers to provide a high quality service.

The autonomy of this work extended to the freedom to connect with multiple clients from diverse industries, sectors and countries. The diversity of clients created an element of variety even with regards to nominally similar tasks. Moreover, online labour platforms provided workers with opportunities to carry out work they were unfamiliar with and provided access to experiences that they would not otherwise have been able to realise. This novelty caused much of the work to be experienced as interesting and enriching; some workers also felt that, as they gained experience, they had opportunities to take on tasks of increasing complexity. That much of the work was stimulating and complex was supported by our survey: 62% agreed that their job involved unforeseen problem solving; and 57% agreed that their job involved solving complex problems.

Men benefited the most in this regard, with 64% reporting solving complex problems, while only 41% of women did so. Male respondents were more likely to undertake tasks that were highly skilled, with 50% undertaking medium- or high-skilled tasks, against just 25% of women. Of those undertaking highly skilled tasks, 66% reported that the work involved solving unforeseen problems and 62% reported that it involved solving complex tasks, compared to 60% and 54% respectively for those doing lower-skilled tasks. This supports Monteith and Giesbert’s (2017) argument that in contexts such as these the gendered division of labour is an important determinant of job quality. Nevertheless, over half of those workers carrying out low-skilled tasks still reported them as involving solving unforeseen problems and complexity. In fact, our interviews suggest that it was only those doing data entry tasks who seemed to find the work unavoidably boring – a type of task common on MTurk.

Despite the experiences of autonomy inherent to algorithmic control, we found that remote gig work could be highly intense. For instance, 54% of our survey respondents said that they had to work at very high speed, 60% worked to tight deadlines and 22% experienced pain as a result of their work. This was due to several reasons related to the organisational form which platform-based rating and ranking technologies enabled. Algorithmic control enabled clients to potentially contract with millions of workers based anywhere in the world. As a result, workers experienced high levels of competition on the platforms for projects/tasks, which meant that it was difficult for them to raise rates. An alternative means of increasing earnings was through completing more tasks. But maximising the number of tasks required that tasks be completed as quickly as possible, thus increasing work intensity. Furthermore, to minimise unpaid downtime and ensure that they had tasks when they wanted to work, workers had to diversify and work for several clients through the platform. This, in turn, required dealing with multiple clients’ deadlines and requirements, again increasing work intensity. Kevin (Kenya; transcription) illustrated the tension between income security and workload:

It’s so insecure… unless you have 10 clients; then you can breathe. But then with 10 clients it means each client has an expectation of a certain workload for you to do… So you can say, ‘I’ll get 10 clients for security’, but then can you satisfy all those 10 clients? You can have three clients, but then when they disappear that’s it.

Finally, the competitive nature of bidding for jobs on the platform also meant that workers promised to meet tighter deadlines than their competitors. As Chris (South Africa; writing) explained: ‘The hard work you’ve got to put in to get it done… It takes quite a lot of effort to produce what I’ve marketed.’

In theoretical terms, this observed work intensity can be explained by how online labour platforms and the platform-based rating and ranking technologies central to them gave rise to an organisational form shaped by the weak structural power of workers vis-a-vis clients. The weak position of workers in bargaining over work and the resulting effects on intensity are also echoed in the survey, with 80% of respondents reporting that the pace of their work was determined by direct demands from clients. SSA respondents slightly more frequently reported experiencing work intensity (6 percentage points more reported working at very high speed and 12 percentage points more reported working to tight deadlines). This is seemingly due to the slightly superior bargaining power of workers from SEA, reflecting the greater demand for them over SSA workers (Kässi and Lehdonvirta, 2016). Nevertheless, high work intensity was common across platforms and countries.

### The flexibility myth and absence of social contact

A further form of autonomy entailed by the platform-based algorithmic control was discretion over place of work. In particular, the ability to work from home was seen as a major benefit, enabling workers to avoid what would otherwise have been long, uncomfortable and costly commutes on poor quality public transport. Control over place of work also facilitated the combination of remote gig work with other forms of work such as studying, domestic work, caring and alternative paid work. But workers’ ability to convert this potential into actual flexibility was often constrained by the lack of affordable alternative places of work. The vast majority of informants worked from home with little variation. There was, however, a downside to this autonomy in that working from home could also lead to social isolation: the loneliness of working without interpersonal contact was an important theme of the interviews with some workers. The importance of the social contact provided by paid work for mental health has long been recognised (Jahoda, 1982; Wood and Burchell, 2018).

Workers valued another dimension of autonomy that this work offered, that of ‘temporal flexibility’ (Wood, 2016). However, an important theme of the interviews was that earning a decent income frequently entailed long working hours. For some workers, these long working hours were spent solely undertaking remote gig work. For example, James (Kenyan; research, data entry, virtual assistant and lead generation) worked 78 hours a week making $3.5 per hour. Others worked long hours as a result of combining gig work with alternative employment, study and caring:

I put in like 40 [hours/week] or even more depending on the magnitude of work… That’s just at night… I work [locally] during the day, so when I go back home I put in five to six hours… then over the weekend… In total it will be like 70… You work 24/7. (Olive, Kenya; data entry, lead generation, website curation, social media marketing, writing)

However, total weekly hours do not tell the full story. As working time was completely unregulated, a few days’ work might be condensed into a single day. As Simon (Kenya; data entry) explained: ‘A client [is] paying me $3.50 an hour. I’m so broke, this is someone who’s ready to give me the money, so why don’t you want 18 hours in one day.’

Working such long hours obviously necessitated unsocial working hours (evenings, nights and weekends) and could be a source of exhaustion: ‘I find that I’m exhausted… you might find yourself working throughout the night… the biggest challenge when you are working online [is] you can find that you are working for so many hours without rest’ (Moses, Nigeria; virtual assistant, research, data entry and lead generation).

These findings highlight that the value that workers place on flexibility (e.g. Berg, 2016; D’Cruz and Noronha, 2016) cannot be uncritically accepted. Agency ‘operates within possibilities, and constraints of social arrangements… in this sense we are both active and passive’ (Jahoda, 1982: 28). Thus it is possible for remote gig workers to value flexibility, even though its realisation is dependent upon plentiful demand (Lehdonvirta, 2018). Such plentiful demand is in reality often a myth for contingent workers based on widely held but largely unrealised assumptions (Henson, 1996).

Moreover, workers’ ability to exert control over flexible working time has been found to be dependent on worker power (Wood, 2016). In this case, the weak structural bargaining power of remote gig workers limited their influence over their working time. For instance, late-night working was common, a consequence of working hours being largely determined by clients. Work had to be completed to meet the clients’ deadlines, communication with clients took place when convenient for clients and new jobs were posted at the times when clients were working: ‘She would send work anytime she wants, [she] doesn’t want to know if you’re busy’ (Diana, Kenya; writing and SEO).^3^

As clients tended to be located in US, UK and Australian time zones, workers were expected to work at night to be in sync with their clients. Fifty-four per cent of our survey respondents said they lost sleep at night while only 28% said they did not. This was a slightly bigger problem among our male respondents (59% agreeing vs. 47% of female respondents). As suggested by Monteith and Giesbert (2017) the division of domestic labour in the household would seem to restrict the ability of some women to work at night to the same extent as men. Working time being largely determined by clients also led to unstructured work patterns, with hours often being irregular and unpredictable. As Kennedy (Kenya; writing, data entry, internet advertising) articulated: ‘Seven days a week. It can be at night, can be during the day, anytime…. Sometimes you don’t have any contracts, so when you have them, you have to work.’

That the client largely determined working time parallels the finding that many workers had little variation in their place of work, despite valuing both these forms of flexibility. This contradiction was vividly expressed by Anita (Kenya; transcription, translation, social media marketing, website curation, research, writing):

I can work at my own convenience… when I want to sleep, I can sleep. Oh, another thing I just remembered, most of the jobs you can get are like from overseas… In [the] USA… it’s time you want to sleep so you have to sacrifice… [by] working in the middle of the night.

### Worker power and competition

As indicated above, the organisational form that remote gig work took was analogous to what Kalleberg (2011) refers to as an open, market mediated employment relationship. In particular, it was marked by high levels of inter-worker competition with few labour protections and a global oversupply of labour relative to demand (Graham et al., 2017a; Graham and Anwar, in press). Workers explained how the instant a job was posted, workers from across the world would enter bids. For example, Joseph (Nigeria; social media advertising and lead generation) explained how: ‘immediately [as] you see an offer being posted… you will see 50 proposals have been submitted’.

Our survey results also suggest an oversupply of labour, with 54% of respondents reporting that there was not enough work available and just 20% disagreeing. Our interviews suggest that this oversupply of labour was in part due to growing global connectivity enabling workers anywhere in the world to connect with clients via online labour platforms, combined with a lack of perceived local labour market alternatives of the same standard for what was a highly educated workforce. Not only did this oversupply of labour make it difficult to get work but constantly experiencing this competitive environment led workers to feel that they could easily be replaced, especially by workers living in another country who were assumed to be willing to work for less money: ‘I’m sure there’s a hundred thousand people out there across the world who could do exactly what I do probably for cheaper as well’ (Ashly, South Africa; writing and research, data capture).

This fear of being undercut was heightened by the fact that workers had no formal job security and thus could have their contract terminated at any time without notice. Online labour platforms framed their workforces as being ‘on-demand’, made up of ‘freelancers’ or ‘independent contractors’ who could be ‘fired on the spot’ by ending their contract without notice. As Amanda (South Africa; writing and data entry) explained: ‘There’s a lot of people out there, if they’re not satisfied with you, they are going to try somebody else… So, they can replace you. This is one of those jobs that you can be replaced.’ This fear of being replaced was also evident among a significant number of survey respondents, with 44% saying they felt easily replaceable against 30% who disagreed.

Since workers not only exercise power relative to employers but also vis-a-vis other workers (Kalleberg, 2011), individual marketplace bargaining power in the form of skills was influential in determining job quality outcomes. One such outcome was pay. Gig work was an important source of income for most of our interview informants and the main source of income for many. Likewise, 73% of our survey respondents reported that remote gig work was an important source of income for their households, and 61% reported that it was their main occupation. Our interview informants were mostly gaining an income sufficient to avoid material hardship. This is supported by our survey data, with the mean weekly income of those paid in USD (*N* = 610) being $165 (σ = $209) from this work (seven outliers of $2000 and over were excluded as improbably large, though not wholly impossible given the unpredictability of gig work). A geographic difference was apparent, with SEA workers’ earnings averaging $181 (*N* = 304) compared to $150 for SSA (*N* = 307). This relates to the fact that 47% of SEA respondents were mainly carrying out high- or medium-skilled tasks compared to 33% for SSA. Across all respondents, those carrying out more skilled work earned on average $44 a week more than those doing low-skilled work. These findings suggest that the quality of national educational systems is important in determining the level of income that remote gig work translates into in a given country.

However, even among survey respondents doing low-skilled work, we find massive income disparities: the income ratio between the 10th and 90th percentile is 1:19 (*N* = 328; excluding respondents earning nothing or above $2000 in the last week). This suggests that other forms of worker power besides skills are also important in determining income. Our interviews suggest this other form of power is reputation, both in the conventional sense of good standing and networks among potential clients, and in the sense of the symbolic power which accrues to some workers as a result of the algorithmic control system outlined above. Work flowed to those workers who had managed to maintain a strong reputation over a long period and were thus known by clients and highly ranked by platform algorithms. A key theme of the interviews was the difficulties faced by workers who lacked strong reputations. They had low incomes, in some cases below their countries’ monthly minimum wages, and a handful survived around the global poverty line of $58 per month. Workers who were struggling financially described how, for example, their pay was not enough to ‘actually survive’ (Helen, South Africa; writing and virtual assistant). Less in-demand workers were also forced to spend longer doing the unpaid work of searching and applying for paid work, which further reduced their potential to earn (our average survey respondent reported spending 16 hours per week looking for work, or 39% of the total time spent on remote gig work).

Workers who were in demand thanks to skills and reputation enjoyed several job quality advantages over others. As they often had multiple clients and could easily replace clients they lost, they felt less pressure towards work intensification, and had labour market and income security (Standing, 1999). The higher income also meant that they could afford to save some of their income and pay for private health insurance, in contrast to workers on low incomes, who did not have any formal social security. As suggested by Kalleberg (2011) and Monteith and Giesbert (2017), this finding highlights the importance of national healthcare systems for job quality.

In summary, the job quality of remote gig work can be explained by its specific organisational form combined with social and environmental conversion factors such as gender relations and educational and health systems. The organisational form experienced by remote gig workers is a product of the confluence of technology that facilitates the detachment of work from place (labour platforms and their platform-based rating and ranking systems) and the power relations, especially individual marketplace bargaining power in the form of skills and reputation.

## Conclusions

Despite conducting a study across diverse national contexts and job types, we find certain key commonalities in the job quality determinants and outcomes of remote gig work. In particular, we find that algorithmic control is central to the operation of online labour platforms. This form of control differs significantly from the Taylorist control often attributed to the extensive use of informational management tools. In contrast to Taylorism, algorithmic management techniques enabled by platform-based rating and ranking systems facilitate high levels of autonomy, task variety and complexity, as well as potential spatial and temporal flexibility. Thus remote gig work is a long way from being an ‘assembly line in the head’ (Bain and Taylor, 2000) or ‘electronic sweatshop’ (Fernie and Metcalf, 1998).

However, while algorithmic control provides remote gig workers with formal control over where they work, workers may have little real choice but to work from home, and this can lead to a lack of social contact and feelings of social isolation. Likewise, despite valuing the potential to control their working hours, most workers had to work intense unsocial and irregular hours in order to meet client demand. The autonomy resulting from algorithmic control can lead to overwork, sleep deprivation and exhaustion as a consequence of the weak structural power of workers vis-a-vis clients. This weak structural power is an outcome of platform-based rating and ranking systems enabling a form of control which is able to overcome the spatial and temporal barriers that non-proximity places on the effectiveness of direct labour process surveillance and supervision. Online labour platforms thus facilitate clients in connecting with a largely unregulated global oversupply of labour.

As suggested by Kalleberg (2011), remote gig workers’ job quality in this open, market-mediated environment is also determined by workers’ marketplace bargaining power in relation to both employers and other workers and thus individual worker resources. In the case of remote gig work, the individual resources that we find to be most important are *skills* and *platform reputation*. Workers lacking these individual resources suffered from low incomes and insecurity. The importance of platform reputation is a consequence of the algorithmic control inherent to online labour platforms. The identification of the ‘symbolic power’ (Thompson, 1991) of platform reputations as an emerging form of marketplace bargaining power is an important contribution of this article. Marketplace bargaining power is a concept developed by Silver (2003) and Wright (2000), neither of whom consider the importance of symbolic forms of power. These findings are likely to become increasingly relevant to the wider world of work as gig economy style algorithmic controls are increasingly adopted within standard employment relationships (Keller, 2017; O’Conner, 2016).

## Notes

1. Kalleberg (2011) points out this growth is really a return to the employment practices which were commonplace in the USA before the 1930s.

2. Lead generation involves searching the internet for contact details, particularly email and telephone numbers, for advertising purposes.

3. Search engine optimisation is a set of techniques to boost the search engine ranking of web content. Common strategies involve creating topically relevant content, and content that search engine algorithms accept to be human – rather than machine – created.
